# Root canal and nasopalatine canal morphology in a Guangxi population: a cone-beam CT study on regional variations

**DOI:** 10.1186/s12903-026-08459-4

**Published:** 2026-04-28

**Authors:** Xinxin Zhu, Xiaohan Huang, Huaming Mai, Xianfang Jiang

**Affiliations:** 1 College of stomatology, Hospital of stomatology, Guangxi Medical University, Nanning, 530021 China; 2 Department of Oral and Maxillofacial Surgery, College &Hospital of Stomatology, GuiLin Medical University, Guilin, 541004 China; 3 Key Laboratory of Oral and Maxillofacial Rehabilitation and Reconstruction, Guangxi Clinical Research Center for Craniofacial Deformity, College & Hospital of Stomatology, Guangxi Medical University, Nanning, 530021 China

**Keywords:** Cone-Beam Computed Tomography, Guangxi, Premolar, Mandibular second molar, Nasopalatine canal

## Abstract

**Objective:**

To characterize the root canal morphology of premolars, mandibular second molars and the anatomical parameters of the nasopalatine canal (NPC) in the Guangxi Zhuang Autonomous Region, China, using cone-beam computed tomography (CBCT), and to explore regional variations.

**Methods:**

This retrospective study analyzed CBCT imaging data from a large population in Guangxi, focusing on the anatomical characteristics of maxillary and mandibular premolars, mandibular second molars, and the NPC. High-resolution CBCT slices and three-dimensional reconstruction techniques were used to quantitatively assess the number, classification, and symmetry of root canals, as well as the linear parameters of the NPC. Statistical analyses were conducted to examine correlations between anatomical variations and factors such as gender, age, and tooth position.

**Results:**

A total of 550 patients were included. The results shown that maxillary first premolars have a high prevalence of multiple canals, with a rate of 86.5%. Females exhibited more complex premolar canal systems than males (ρ = 0.180, *P* < 0.001). C-shaped canals in mandibular second molars occur at a rate of 39.8%, with 28.0% showing bilateral symmetry. It was negatively correlated with the presence of multiple canals in premolars, as evidenced by a correlation coefficient of ρ=–0.141 (*P* < 0.001). NPCs were predominantly of the single-canal type (75.6%). Canal diameter increased with age (ρ = 0.227, *P* < 0.001) and was larger in males (*P* < 0.001).

**Conclusions:**

The Guangxi population exhibits substantial premolar canal variation and a notable prevalence of C-shaped canals in mandibular second molars, indicating distinct regional anatomical patterns. Preoperative CBCT evaluation is essential for endodontic and implant procedures. The age-related increase in NPC diameter provides important anatomical considerations for anterior maxillary implant safety.

## Introduction

Advancements in modern root canal treatment techniques have revealed the complex anatomy of teeth and root canal systems. Accurate identification and effective management of anatomical variations in the root canal system are fundamental to ensuring the long-term success of root canal treatment (RCT), and reducing complications such as missed canals and iatrogenic perforations [1].The investigation of root canal morphology has advanced significantly with the introduction of technology [2]. Compared with two-dimensional imaging, three-dimensional imaging can precisely reconstruct the spatial trajectory, bifurcation angles, and local anatomical features of root canals [3, 4]. Micro-CT offers sub-10 μm resolution that reliably quantifies isthmus and lateral-canal morphology, but it cannot be used in clinical medical practice due to elevated radiation dose, high operating cost, time-consuming data processing, poor cost-effectiveness, and safety concerns. Additionally, it is restricted to ex vivo investigations and lacks in vivo applicability [5]. Cone-beam computed tomography (CBCT) delivers 3-D images at a clinically acceptable dose and therefore remains the most common assessment tool of root-canal anatomy and obturation quality [6, 7].

Premolars and mandibular second molars are considered high-risk teeth in root canal therapy owing to the complexity of their root canal systems [8, 9]. Anatomically, premolar root canals exhibit considerable variation in number and may contain complex features, such as isthmuses. Similarly, mandibular second molars frequently present with a C-shaped root canal system [10], characterized by significant morphological diversity across individuals and ethnic groups. Vertucci’s classification [11], proposed by Vertucci in 1984, is the most frequently employed descriptive system for root canal anatomy in current clinical and imaging research. It categorizes the communication patterns of root canals from the floor of the pulp chamber to the apical foramen into eight types (I–VIII), enhancing comparability and communication between research and clinical practice. The nasopalatine canal (NPC) represents another critical anatomical landmark in the anterior maxilla. Recent CBCT-based morphometric studies [12, 13] have shown that NPC length and maximum diameter positively correlate with the bucco-lingual diameter of premolar sockets, thereby affecting the probability of root canal bifurcation and the number of canals present. Consequently, accurate radiological assessment of NPC morphological characteristics and anatomical variations is essential to prevent complications and ensure appropriate clinical practices in local anesthesia, maxillary surgery, implant placement, and orthodontic tooth movement [14, 15].

Ancient Guangxi populations demonstrated deeply lineages characterized by highly intricate hybrid genetic structures. In contrast to the simple continuation of a singular lineage, the Guangxi population exhibited significant genetic heterogeneity [16]. Despite this rich genetic diversity, a notable gap exists in anatomical studies of the Guangxi Zhuang Autonomous Region, particularly regarding indigenous residents with distinct genetic backgrounds [17]. To address this gap, we conducted a comprehensive evaluation of root canal morphology in premolars and mandibular second molars, along with the anatomical parameters of the nasopalatine canal (NPC), in the Guangxi population. Our analysis examined variations according to age, sex, laterality, and bilateral symmetry. Additionally, we quantified the morphological classification and key metrics of the NPC to elucidate the clinical relevance of its anatomical variations for site-specific surgical planning. This regional morphological database provides essential anatomical guidance for clinical dentistry and risk assessment in Guangxi and serves as a foundation for cross-regional comparative studies of dental anatomy among East Asian populations.

## Materials and methods

### Data collection

A total of 6050 CBCT images including 4400 premolars and 1100 mandibular second molars and 550 NPC were obtained from 550 patients born in Guangxi province who required radiographic examination by CBCT as part of their dental treatment at Stomatological Hospital of Guangxi Medical University, between January and October 2021. Demographic and clinical information, including ethnicity, sex, age, and treatment history, was extracted from medical records. This study received ethical approval from the Ethics Committee of the College & Hospital of Stomatology, Guangxi Medical University (Approval No. GXMUHS-KY2026-001-CS). The requirement for informed consent was waived by the ethics committee due to the retrospective nature of the study and the use of de-identified data.

### Demographic characteristics

Among the 550 study participants, 257 were male (46.73%), and 293 were female (53.27%). The age range was 16 to 73 years, with an average age of 33.93 ± 13.14 years. The sample distribution covered young, middle-aged, and elderly groups, providing good population representativeness.

### Inclusion criteria

 (1) Age ≥ 16 years with complete root development (2). Good systemic health with no history of bone metabolic disease, jaw tumors, or systemic syndromes (3). Intact target teeth (bilateral premolars, mandibular second molars, and anterior maxillary region) without severe carious destruction of canal morphology or extensive metal restorations that would produce scatter artifacts (4). Target teeth without prior root canal treatment or apical surgery.

### Exclusion criteria

 (1) Severe tooth deformities or developmental anomalies (e.g., dens invaginatus, fused teeth, microdontia) (2). CBCT images of inadequate quality due to motion artifacts, insufficient contrast, or field-of-view obstruction that precluded clear identification of target structures. After evaluation, 511 of 1061 CBCT scans (48.2%) were excluded.

### Imaging acquisition and parameters

All scans were performed by an experienced radiologic technologist using a KaVo 3D eXam CBCT system. Scan parameters: (1) Voltage and current: 120 kV, 7 mA (2). Acquisition time: 26.9 s (3). Voxel size: 0.25 mm (4). Field of view (FOV): full dentition covering anterior maxilla, entire arch, and periapical tissues.

### Imaging assessment, measurement, and anatomic classification criteria

An experienced oral and maxillofacial radiologist performed 3D reconstruction using dedicated software. After all samples were measured, 30% of the samples were randomly selected and re-measured by the same examiner two weeks later. The results demonstrated high test-retest reliability (ICC > 0.9, *p* < 0.001). Multiplanar evaluation in the sagittal, coronal, and axial planes enabled classification and measurement of the following anatomical structures.

### Criteria for multi-canal premolars

**Fig. 1:**
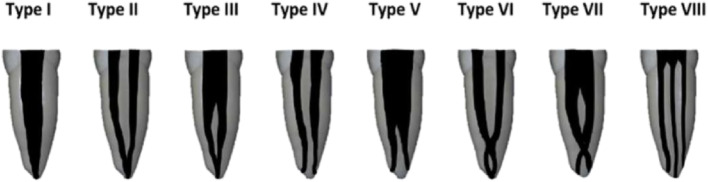
Vertucci’s classification of root canal configuration

Root canal configuration was classified in Vertucci’s classification (Fig. 1) [11]: Type Ⅰ was considered as 1 canal, Type Ⅱ to Ⅶ were considered as 2 canals, and Type Ⅷ was considered as 3 canals. Multi-canal premolars were identified according to literature criteria [18]: (1) Maxillary first premolars, normally bilateral, were defined as multi-canal variations when > 2 canals were present (2). Maxillary second and mandibular premolars (first and second), normally single-canal, were defined as multi-canal systems when ≥ 2 canals were present.

### C-shaped canals in mandibular second molars

**Fig. 2:**
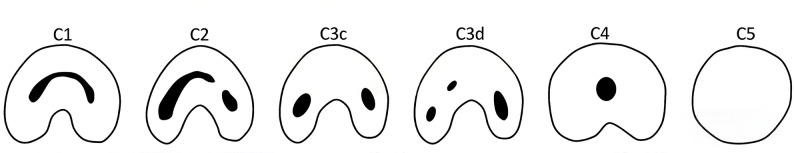
Fan et al. classification of C-shaped canal configuration

C-shaped canals were identified following Fan et al.‘s classification [19–21]: (1) Morphology: the root and canal system exhibited a continuous “C”-shaped arc in cross-section, or a non-continuous arc with isthmus connections (2). Assessment: full-length observation at cervical, middle, and apical segments. Only teeth showing fused root characteristics on at least one level were classified as C-shaped canals(Fig. 2).

### Nasopalatine canal (NPC) anatomic classification and measurement

**Fig. 3:**
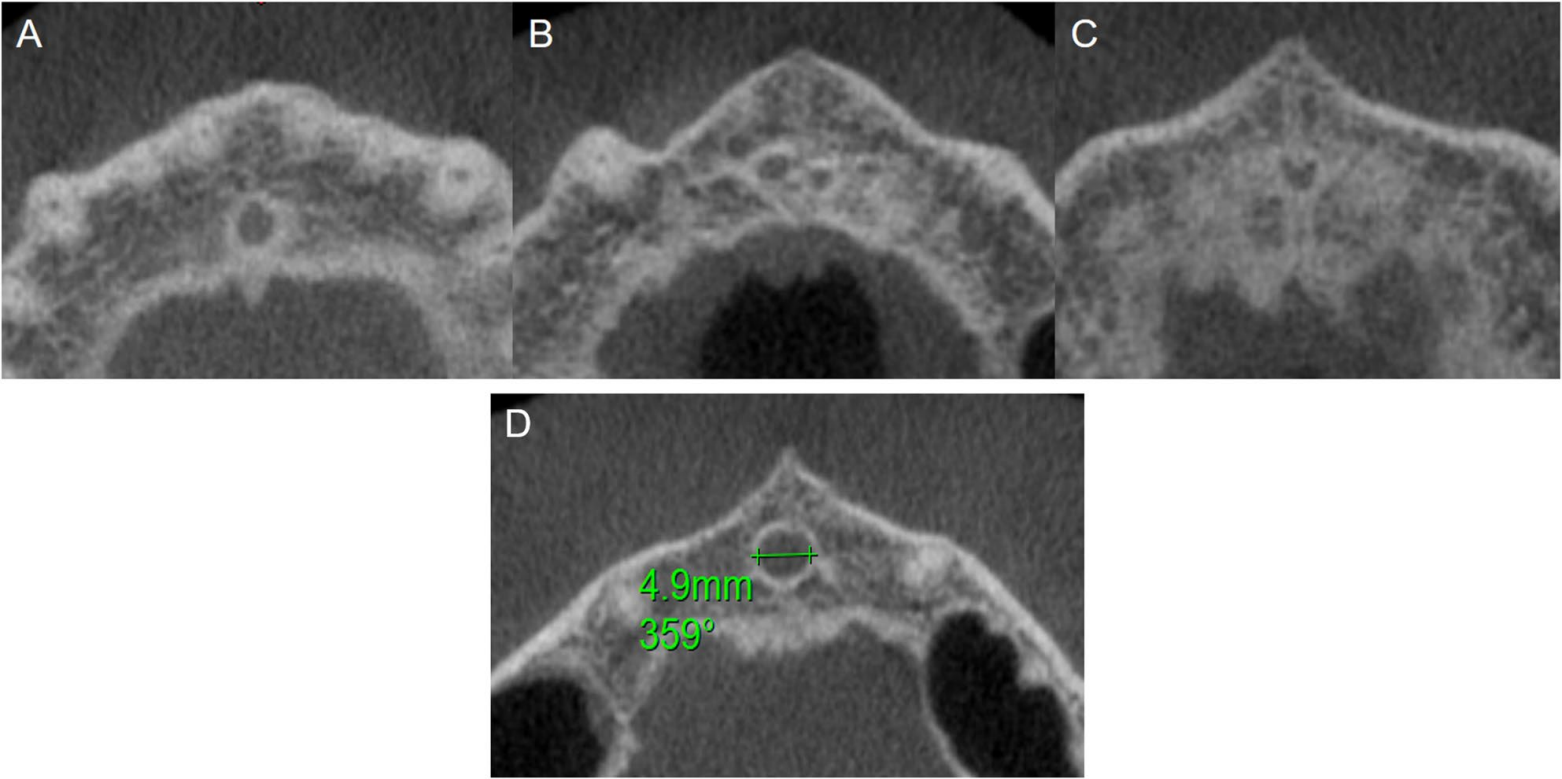
The nasopalatine canal on CBCT images. **A **Single. **B **Double. **C **Y-shaped. **D **the measurement of the NPC

The NPC, a critical midline maxillary landmark, was evaluated using Bornstein et al.‘s classification [22]: (1) Morphologic types: (a) Single: one canal from nasal floor to palate without branching. (b) Double: two parallel canals separated by a bony septum. (c) Y-shaped: two nasal openings converging to a single palatal exit (2). Maximum diameter: measured on axial images at the widest cross-section as the greatest distance between inner canal walls(Fig. 3).

### Statistical analysis

Statistical analysis was performed using SPSS version 31.0 (IBM Corp., Armonk, NY, USA). Normality was tested by Kolmogorov-Smirnov test. Continuous variables with normal distribution were expressed as mean ± standard deviation (SD). For non-normally distributed continuous variables, data are presented as median (interquartile range, IQR). Between-group comparisons were made using independent samples t-test for two groups and one-way ANOVA for three or more groups. Categorical data were presented as percentages (%) and analyzed using chi-square test. Spearman rank analysis was used for correlation analysis. *P* < 0.05 was considered statistically significant.

## Results

### Distribution characteristics of premolar root canal numbers

This study evaluated 1,100 CBCT images of four premolar types and revealed statistically significant differences in the number of root canals among the different premolar groups (Table 1).

**Table 1:** Distribution of Root Canal Numbers in Premolars

Tooth position	Total	1 canal	2 canals	3 canals
Maxillary first premolar	1100	137(12.5%)	951(86.5%)	12(1.1%)
Maxillary second premolar	1100	714(64.9%)	381(34.6%)	5(0.5%)
Mandibular first premolar	1100	905(82.3%)	187(17.0%)	8(0.7%)
Mandibular second premolar	1100	1097(99.7%)	3(0.3%)	0(0.0%)

#### Maxillary first premolars

Maxillary first premolars most commonly presented with 2 root canals (86.5%, 951/1,100), followed by 1 root canal (12.5%, 137/1,100) and 3 root canals (1.1%, 12/1,100). Among all 1,100 maxillary first premolars, 2 root canals were observed in 470 male teeth (42.7%, 470/1,100) and 481 female teeth (43.7%, 481/1,100) (Table 2). The gender-related differences in prevalence were not statistically significant (*P* > 0.05). Notably, significant bilateral symmetry in morphology was observed (Table 3).

**Table 2:** Gender Distribution of Root Canal Numbers in Maxillary First Premolars (*n* = 1,100)

Root canals	Male	Female	*P*
1	35(3.2%)	102(9.3%)	*< 0.05*
2	470(42.7%)	481(43.7%)	
3	9(0.8%)	3(0.3%)	

**Table 3:** Bilateral Symmetry Analysis of Maxillary First Premolars (*n* = 550)

	1 canal	2 canals	3 canals
Bilateral symmetry	59(10.7%)	463(84.2%)	3(0.5%)

#### Maxillary second premolars

In contrast to maxillary first premolars, maxillary second premolars were most frequently characterized by a single root canal (64.9%, 714/1,100), while multiple canals were observed in 35.1% of cases.

#### Mandibular first premolars

Among the 1,100 mandibular first premolars evaluated, 905 (82.3%, 905/1,100) had a single canal (1R1C), whereas 17.7% (195/1,100) exhibited 2 or more canals or more complex configurations.

#### Mandibular second premolars

Among the four premolar types, mandibular second premolars exhibited the highest degree of anatomical consistency, with a single canal prevalence of 99.7% (1,097/1,100) and a negligible incidence of canal bifurcation or multiple canals (0.3%, 3/1,100).

### Morphological characteristics of C-shaped root canals in mandibular second molars

Comprehensive evaluation of mandibular second molars in 550 patients elucidated the incidence and distribution characteristics of C-shaped root canal morphology as presented in Tables 4 and 5.

**Table 4:** Incidence of C-shaped root canals in mandibular second molars

	*n*	Cases	Prevalence	χ²	*P*
Gender					
Male	257	83	32.3%	11.392	*< 0.001*
Female	293	136	46.4%		
Age					
< 30yr	255	107	42.0%	1.001	*0.606*
30-50yr	221	85	38.5%		
> 50yr	74	27	36.5%		

**Table 5:** Bilateral incidence of C-shaped root canals in mandibular second molars (*n* = 550)

	Cases	Prevalence (%)
C-shaped canals	219	39.8%
Unilateral occurrence	65	11.8%
Bilateral occurrence	154	28.0%

#### Overall incidence and gender differences (table 4)

Among the 550 CBCT scans, 219 patients (39.8%, 219/550) exhibited C-shaped root canal structures, including unilateral or bilateral occurrences. Notably, females demonstrated a significantly higher detection rate (46.4%, 136/293) compared to males (32.3%, 83/257) (χ² = 11.392, *P* < 0.001). This finding indicates substantial gender heterogeneity in the anatomical variations of mandibular second molars.

#### Age-related distribution characteristics (table 4)

Comparison of C-shaped root canal detection rates across different age groups (young: <30 years, middle-aged: 30–50 years, elderly: >50 years) revealed no statistically significant differences (*P* = 0.606). This suggests that C-shaped root canals, as developmental anatomical features, remain stable throughout the lifecycle after root development completion.

#### Bilateral symmetry distribution (table 5)

Among the 219 patients with C-shaped root canals, bilateral symmetrical occurrences were predominant at 28.0% (154/550), while unilateral C-shaped root canals accounted for 11.8% (65/550). Clinically, this indicates that detection of a C-shaped root canal in one mandibular second molar suggests a high probability of similar anatomical variations in the contralateral tooth.

### Anatomical morphological evaluation of the nasopalatine canal (NPC)

Based on the classification criteria established by Bornstein et al. [21], this study detailed the morphological distribution and linear measurement parameters of the nasopalatine canal (Table 6).

**Table 6:** Morphological classification and maximum diameter of the nasopalatine canal

Morphologic type	Cases	Male	Female	χ^2^/F	*P*
Single canal	416	204(79.4%)	212(72.4%)	3.675	*0.159* ^*a*^
Double canal	31	12(4.7%)	19(6.5%)		
Y-shaped	103	41(16.0%)	62(21.2%)		
Maximum diameter (mm)	3.7(1.4)	3.9(1.3)	3.6(1.4)	7.834b	0.005b

a Chi-square test

b Independent T test

#### Morphological classification distribution

Among the 550 study samples, the single-canal type was most prevalent, with a detection rate of 75.6% (416/550), followed by the Y-shaped canal (dual nasal openings converging to a single palatal opening) accounting for 18.7% (103/550), and the double-canal type (parallel independent ducts) being the least common at 5.6% (31/550). Chi-square analysis revealed no statistically significant differences in nasopalatine canal morphology between genders (χ² = 3.675, *P* = 0.159).

#### Maximum diameter of the nasopalatine canal

The maximum diameter was measured at the widest point on axial CBCT images. The overall mean maximum diameter was 3.7(1.4) mm. Independent t-test analysis revealed statistically significant gender-related differences (t = 7.834, *P* = 0.005), with males exhibiting a larger mean diameter (3.9(1.3) mm) compared to females (3.6(1.4) mm).

### Correlation analysis of multidimensional anatomical indices

Spearman’s rank-order correlation (ρ) was used to evaluate correlations among variables including premolar root canal counts, C-shaped canal prevalence, NPC morphometric parameters, age, and sex (Table 7).

**Table 7:** Correlation analysis of multidimensional anatomical parameters

Variable	C-shaped canal	*P*	Premolar multi-canal	*P*	NPC maximum diameter		*P*		Age		*P*	Gender	*P*
C-shaped canal	1.000	-	-0.141	< 0.001	-0.037		0.222		-0.063		0.038	-0.144	< 0.001
Premolar multi-canal	-0.141	< 0.001	1.000	-	-0.058		0.055		0.006		0.850	0.180	< 0.001
NPC maximum diameter	-0.037	0.222	-0.058	0.055	1.000^b^		-		0.227		< 0.001	0.107	< 0.001
Age	-0.063	0.038	0.006	0.850	0.227		< 0.001		1.000		-	0.157	< 0.001
Gender	-0.144	< 0.001	0.180	< 0.001	0.107		< 0.001		0.157		< 0.001	1.000	-

### Root canal system correlation

A negative correlation was observed between C-shaped canal prevalence in mandibular second molars and multiple root canal occurrence in premolars (ρ = −0.141, *P* < 0.001). This negative correlation suggests that as the prevalence of C-shaped canals in mandibular second molars increases, the occurrence of multiple root canals in premolars tends to decrease. This may indicate a developmental dichotomy between the confluent pattern of molar root systems (C-shaped canals) and the separated pattern of premolar root systems (multiple canals).

### Gender and root canal variations

A positive correlation was found between multiple root canal occurrence in premolars and gender (ρ = 0.180, *P* < 0.001). Morphological analysis revealed that females demonstrated a significantly higher prevalence of anatomical variations at specific tooth positions.

### NPC morphological changes

A positive correlation was observed between age and NPC maximum diameter (ρ = 0.227, *P* < 0.001), indicating that NPC diameter tends to increase with advancing age. Additionally, significant gender-related differences were observed in this parameter (ρ = 0.107, *P* < 0.001).

## Discussion

### Morphological complexity and gender variations in premolar root canal systems

Premolars have unusual root and canal anatomy. This increases the risk of endodontic failure if additional canals are undiagnosed [23]. Besides, enlargement of a curved root canal often results in loss of working length, incomplete removal or extrusion of the root canal content and unwanted alterations in the canal shape, such as transportation (stripping, zipping, elbow formation), ledge formation and perforation [24, 25]. Understanding root canal morphology is essential for successful endodontic treatment.

This study systematically analyzed 4400 premolars from the Guangxi population. The results showed that the most common anatomy of maxillary first premolars was two canals (86.5%), and females exhibited a higher prevalence of this configuration compared to males. Maxillary contralateral first premolars demonstrated a high degree of symmetry in root and canal anatomy, over 90% of pairs shared identical Vertucci types, as reported in Saudi and Turkish Cypriot CBCT cohorts [26, 27]. This morphology differs significantly from other premolar positions. Therefore, exploration for additional canals is necessary during treatment of maxillary first premolars.

Single canal morphology was predominant in maxillary second premolars (64.9%), mandibular first premolars (82.3%), and mandibular second premolars (99.7%). This distribution is consistent with established literature reporting the dominance of single canals in premolars: Saudi CBCT data documented single-canal rates of 90% and 95% in mandibular first and second premolars, respectively [28]; a global review reported 91% for the second premolar [29], and an anatomical study in Poland found 89% for the first premolar [30].

Correlation analysis confirmed a positive correlation between premolar root canal number and gender (ρ = 0.180, *P* < 0.05), indicating a higher risk of anatomical variations in females. This finding contradicts a recent multinational study [31] reporting higher multiple canal incidence in male maxillary first premolars, and a Turkish study [32] observing opposing sex-based canal patterns. Genetic factors and racial variations might contribute to disparities in the incidence of root number and canal number among human populations. In light of the high prevalence of intricate configurations, a meticulous exploration of supplementary canals is justified during endodontic therapy.

### Epidemiological characteristics and regional heterogeneity of c-shaped canals in mandibular second molars

C-shaped canal systems typically feature a thin isthmus connecting the main canals in the coronal and middle thirds, appearing as “two canals linked by a delicate bridge” in cross-section. This anatomy significantly complicates debridement and obturation [33]. C-shaped molars carry a high risk of strip perforation due to their delicate anatomy [34]. Furthermore, the absence of 3D CBCT evaluation hinders the identification of critical danger zones [35]. This oversight creates stress concentration points in curved canals, precipitating strip perforations and contributing to treatment failure.

Systematic reviews have shown that the prevalence of C-shaped canal configuration in mandibular second molars ranges from 12% to 17% [36], significantly higher than other molars (*P*<0.05), with higher rates observed in East-Asian populations (44%) and in females [37, 38]. Statistical test revealed a significant variation between the Asian and non-Asian population. Compared to Western populations (6.8%–11.3%) [38], East Asian and Chinese populations exhibited higher susceptibility (29%–35.56%) [39]. Domestic comparative studies showed higher incidence rates in mid-to-low altitude regions like Shandong and Sichuan (32.84%–41.64%) [9, 40, 41] compared to high-altitude regions like Xinjiang (29.17%) [42], with Han populations exhibiting higher rates than ethnic minorities.

In this study, the prevalence of C-shaped canals in the Guangxi population was 39.8%, with a higher prevalence in females (*P* < 0.001). This figure falls within the range previously described for East-Asian cohorts (29–45%) [21, 43].

Moreover, the study confirmed a high symmetry rate (28.0%). The prevalence of C-shaped canals in mandibular second molars demonstrates a negative correlation with multi-canal premolars (ρ = -0.141, *P* < 0.05). Clinicians should routinely evaluate contralateral teeth in suspected cases and utilize preoperative CBCT to identify configuration changes along the root segments.

### Morphological characteristics of the NPC and clinical relevance to implant restoration

The NPC exhibits marked morphometric variability across populations, dimensions vary with age, sex, edentulism, and ethnicity [44, 45].

A research on coronal reconstructions reveals that Y-shaped NPC predominate in other populations (63.3%) [46], contrasting with the single-canal(75.6%) dominance observed in our study. This discrepancy substantiates a population-specific anatomical signature in Southern Chinese (Guangxi) individuals. It highlights ethnic variations in dental morphology.

This study confirmed a positive correlation between the maximum diameter of the NPC and age (ρ = 0.227, *P* < 0.05). The positive correlation between NPC diameter and age aligns with reports of age‑related volumetric enlargement after 50 years, suggesting clinically significant remodeling rather than a passive consequence of alveolar crest reduction [47]. This phenomenon should not merely be viewed as passive bone resorption but rather understood as age-related compensatory remodeling. With age increasing, coupled with tooth wear and physiological reduction of the alveolar ridge crest, peri-ductal bone resorption occurs, leading to relative expansion of the canal diameter [48].

The NPC diameter exhibited significant sexual dimorphism (male > female, *P* < 0.001). Compared with females, males exhibit larger diameters at the nasal and oral openings of the NPC, greater buccal bone width anterior to the canal, and longer canal length [49]. It implied a narrower “safe zone” in the incisal region for men and the need for individualized CBCT‑based planning [45]. This difference stems not only from genetic background but also deeper factors such as sex hormone receptor distribution and long-term functional loading. Higher bone turnover rates and stronger masticatory muscle stimulation in males collectively promote physiological widening of the canal lumen [50, 51].

Since NPC morphology is regulated by both age and sex, “age-related expansion” does not occur uniformly but rather presents individualized risk characteristics [52]. Preoperative CBCT should be considered to define three-dimensional safety margins and to avoid “danger zones” during implant placement [53].

### Hypothesis

Root morphogenesis is driven by epithelial-mesenchymal interactions and regulated through conserved pathways, including Wnt, Shh, and Bmp [54]. The Hertwig’s epithelial root sheath (HERS) functions as a signaling center that controls tooth root size, shape, and number [55]. During the developmental window of the dental arch, conserved morphogens control HERS behavior based on dosage and timing, influencing canal branching or fusion across segments [56]. Current theories propose that C - shaped canals result from focal HERS disruptions or delayed fusion on the root surface [57], while multiple canals in premolars are due to HERS epithelial hyper - branching and septum folding in specific regions [58]. 

The weak negative correlation (ρ = -0.141, *P* < 0.05) between the prevalence of C-shaped canals in mandibular second molars and the occurrence of multiple root canals suggests but does not establish a developmental trade-off. We therefore speculate that the limited supply of morphogen during the period of dental - arch development might bias HERS towards fusion or maintenance in posterior segments. Meanwhile, it could possibly constrain excessive branching or folding in anterior segments [59]. This hypothesis, which may align with the notion that “different tooth positions within the same dental arch compete for limited morphogen signals” [60–62] as suggested by some studies, still requires validation through molecular or genetic perturbation experiments. No causal claims are made in this study.

Our root and root canal morphology map provides a regional baseline for preoperative identification of high-variability teeth. The 3D complexity of the root canal system affects the completeness of debridement and obturation. Incorrect anatomical assumptions leave untouched areas, leading to uncleaning and incomplete fills [63]. In curved canals or parallel canal configurations, warm vertical or other thermoplasticized techniques can improve gutta-percha adaptation. Combining flowable sealers with activated irrigation can enhance sealing and debridement of irregularities [64]. When 2D imaging cannot determine canal number or trajectory, limited-FOV CBCT can provide 3D confirmation and help optimize access and technique, following ALADAIP and indication-based dose optimization [65].

On 2D radiographs, internal root resorption appears as a canal-centered radiolucency and may mimic anatomic variants or external resorption. Limited-FOV CBCT can define lesion nature, extent, and perforation status and guide management [66]. Our regional baseline of common configurations and rare variants provides a reference to distinguish normal variants from pathology and reduce misdiagnosis. If premolar and molar morphology remains unclear, escalate to CBCT under dose-optimization principles [64].

In future research, it is worth considering conducting testable modeling and cross-regional comparisons based on the existing CBCT database of the Guangxi population. We may be able to propose a path from regional data to clinical tools. Firstly, carry out standardized 3D measurements and radiomic feature extraction for the root canal and NPC [67]. Then, use these measurement results and features as unified inputs for a multidimensional prediction model [68]. Combining the automatic segmentation and 3D modeling algorithms that have been implemented on CBCT can provide technical support for the objective and consistent extraction of features from large samples [69].

One limitation of our study is that the use of a single examiner may introduce operator bias, which could skew the results. To mitigate this limitation in future studies, employing multiple examiners and implementing a standardized protocol for assessment. Besides, the absence of a power analysis in this retrospective study is another limitation. To address this, future research should incorporate a priori power calculations to ensure adequate sample sizes and enhance the robustness of the statistical analyses. Despite these limitations, the study stands as the first to elucidate the partial root canal morphological characteristics specific to the Guangxi population, filling a critical knowledge gap in the dental literature.

## Conclusions

This study utilized CBCT imaging to systematically quantify the anatomical characteristics of the premolars, mandibular second molars, and NPC in the Guangxi population. The results demonstrate that the root canal system in this population exhibits unique complexity and variation patterns, and the age-related increase in NPC diameter provides important anatomical considerations for anterior maxillary implant safety. Future research should integrate multi-omics technologies and AI-driven imaging techniques to develop multidimensional predictive models, validated through multicenter randomized controlled trials (RCTs).

## Data Availability

All data generated or analysed during this study are included in this published article.

## References

[1] Ahmed HMA, Ibrahim N, Mohamad NS, Nambiar P, Muhammad RF, Yusoff M, Dummer PMH. Application of a new system for classifying root and canal anatomy in studies involving micro-computed tomography and cone beam computed tomography: Explanation and elaboration. Int Endod J. 2021;54(7):1056–82. 33527452 10.1111/iej.13486

[2] Kolarkodi SH. The importance of cone-beam computed tomography in endodontic therapy: A review. Saudi Dent J. 2023;35(7):780–4. 38025595 10.1016/j.sdentj.2023.07.005 PMC10658366

[3] Kaur K, Saini RS, Vaddamanu SK, Bavabeedu SS, Gurumurthy V, Sainudeen S, et al. Exploring Technological Progress in Three-Dimensional Imaging for Root Canal Treatments: A Systematic Review. Int Dent J. 2025;75(2):1097–112. 39030097 10.1016/j.identj.2024.05.014 PMC11976478

[4] A. S. Artificial Intelligence in Endodontics: A Scoping Review. Iran Endod J. 2024;19(2):85–98. 38577001 10.22037/iej.v19i2.44842 PMC10988643

[5] Karobari MI, Arshad S, Noorani TY, Ahmed N, Basheer SN, Peeran SW, et al. Root and Root Canal Configuration Characterization Using Microcomputed Tomography: A Systematic Review. J Clin Med. 2022;11(9):2287. 35566414 10.3390/jcm11092287 PMC9099997

[6] Nambiar P, Pang K, Raja K. A comparative study on mandibular premolar root canal morphology employing cone-beam computed tomography and microcomputed tomography imaging. J Conservative Dentistry. 2022;25(2):173–8. 10.4103/jcd.jcd_606_21 PMC9205352 35720827

[7] Rodrigues CT, Jacobs R, Vasconcelos KF, Lambrechts P, Rubira-Bullen IRF, Gaêta-Araujo H, et al. Influence of CBCT-based volumetric distortion and beam hardening artefacts on the assessment of root canal filling quality in isthmus-containing molars. Dentomaxillofacial Radiol. 2021;50(5):20200503. 10.1259/dmfr.20200503 PMC8231682 33400563

[8] Nassani MZ, Kay EJ. Tooth loss - an assessment of dental health state utility values. Commun Dent Oral Epidemiol. 2011;39(1):53–60. 10.1111/j.1600-0528.2010.00563.x 20690933

[9] Zheng Q, Zhang L, Zhou X, Wang Q, Wang Y, Tang L, et al. C-shaped root canal system in mandibular second molars in a Chinese population evaluated by cone-beam computed tomography. Int Endod J. 2011;44(9):857–62. 21599707 10.1111/j.1365-2591.2011.01896.x

[10] Vasileva VR, Borisova-Papancheva T, Zaneva-Hristova D, Georgieva S, Miteva A-M. C-shaped Canal in Second Mandibular Molar: A Case Report. Cureus. 2025;17(4):e81784. 40330375 10.7759/cureus.81784 PMC12053441

[11] FJ V. Root canal anatomy of the human permanent teeth. Oral Surg Oral Med Oral Pathol. 1984;58(5):589–99. 6595621 10.1016/0030-4220(84)90085-9

[12] Beshtawi KR. Morphology and dimensions of the nasopalatine canal in Palestinian patients assessed using cone beam computed tomography. Sci Rep. 2025;15(1):13248. 40247015 10.1038/s41598-025-97392-7 PMC12006304

[13] Jang M, Yu S-K, Lim S-H, Jih M-K, Jeong S. Radiologic study of the distance between the maxillary central incisor root and the incisive canal according to skeletal malocclusion classification. Sci Rep. 2024;14(1):16875. 39043917 10.1038/s41598-024-68014-5 PMC11266507

[14] Bahşi I, Orhan M, Kervancıoğlu P, Yalçın ED, Aktan AM. Anatomical evaluation of nasopalatine canal on cone-beam computed tomography images. Folia Morphol (Warsz). 2019;78(1):153–62. 30009362 10.5603/FM.a2018.0062

[15] Al-Rokhami RK, Gu H, Dang X, Li Z, Elayah SA, Zhao X, Sakran KA. 3D analysis of morphological changes, maxillary central incisor–incisive canal relationship, and root resorption in subjects with maxillary incisors protrusion who underwent non-extraction fixed appliance therapy considering demographic and skeletal factors: A retrospective study. Int Orthod. 2025;23(3):100993. 40106940 10.1016/j.ortho.2025.100993

[16] Wan W, Huang H, Hu C, Zhang X, Zhou Q, Yang M, et al. Ancient genomes in Southwest China revealed genetic interactions among diverse populations in the historical period. BMC Biol. 2025;23(1):174. 40597302 10.1186/s12915-025-02299-4 PMC12211951

[17] Couso-Queiruga E, Pedrinaci I, Avila-Ortiz G, Chappuis V, Barboza EP, Petersen RL, et al. Characterization of the anterior maxillary region for immediate implant placement: A radiographic cross-sectional study. J Prosthet Dent. 2026;135(1):78–85. 40102166 10.1016/j.prosdent.2025.02.025

[18] Erkan E, Olcay K, Eyüboğlu TF, Şener E, Gündoğar M. Assessment of the canal anatomy of the premolar teeth in a selected Turkish population: a cone-beam computed tomography study. BMC Oral Health. 2023;23(1):403. 37337200 10.1186/s12903-023-03107-7 PMC10280818

[19] Evan A, Lynn DDS, State Dent. J. 2006;72(6):32–4. 17203853

[20] Darlene C, Melton D, Keith V, Krell DDS, MA MS, Fuller MW. DDS, MS. Anatomical and Histological Features of C-Shaped Canals in Mandibular Second Molars. J Endod. 1991;17(8):384–8. 1809802 10.1016/S0099-2399(06)81990-4

[21] Khawaja S, Alharbi N, Chaudhry J, Khamis AH, Abed RE, Ghoneima A, Jamal M. The C-shaped root canal systems in mandibular second molars in an Emirati population. Sci Rep. 2021;11(1):23863. 34903785 10.1038/s41598-021-03329-1 PMC8668958

[22] Al-Ghurabi ZH, Al-Bahrani ZM. Radiographic Assessment of Nasopalatine Canal Using Cone Beam Computed Tomography. J Craniofac Surg. 2020;31(1):e4–6. 31403513 10.1097/SCS.0000000000005805

[23] Noguchi I, Suzuki K, Yoshida T, Yamamoto Y, Ono M, Yamazaki Y, et al. Development of Innovative Contra-angle Handpiece Device with Piston Movement for Root Canal Preparation. J Endod. 2021;47(4):641–7. 33434564 10.1016/j.joen.2020.12.018

[24] Chaniotis A, Ordinola-Zapata R. Present status and future directions: Management of curved and calcified root canals. Int Endod J. 2022;55(S3):656–84. 35106792 10.1111/iej.13685

[25] Harada T, Harada K, Nozoe A, Tanaka S, Kogo M. A Novel Surgical Approach for the Successful Removal of Overextruded Separated Endodontic Instruments. J Endod. 2021;47(12):1942–6. 34481831 10.1016/j.joen.2021.08.012

[26] Celikten B, Orhan K, Aksoy U, Tufenkci P, Kalender A, Basmaci F, Dabaj P. Cone-beam CT evaluation of root canal morphology of maxillary and mandibular premolars in a Turkish Cypriot population. BDJ Open. 2016;2(1):15006. 29607060 10.1038/bdjopen.2015.6 PMC5831013

[27] Mirah MA, Bafail A, Baik A, Abu zaid B, Hakeem M, Ghabbani H. Root Canal Morphology of Premolars in Saudis. Cureus. 2023;15(9):e45888. 37885565 10.7759/cureus.45888 PMC10599201

[28] Mustafa M, Karobari MI, Al-Maqtari AAA, Abdulwahed A, Almokhatieb AA, Almufleh LS, et al. Investigating root and canal morphology of anterior and premolar teeth using CBCT with a novel coding classification system in Saudi subpopulation. Sci Rep. 2025;15(1):4392. 39910098 10.1038/s41598-025-86277-4 PMC11799371

[29] Cleghorn B, Christie W, Dong C. The Root and Root Canal Morphology of the Human Mandibular Second Premolar: A Literature Review. J Endod. 2007;33(9):1031–7. 17931927 10.1016/j.joen.2007.03.020

[30] Różyło1 TK. MM, I. Różyło-Kalinowska1, F. Burdan2. Morphology of root canals in adult premolar teeth. Folia Morphol (Warsz). 2008;67(4):280-5. 19085869

[31] Pertek Hatipoğlu F, Magat G, Karobari MI, Madarati AA, Tulegenova I, Hatipoğlu Ö, et al. Root and canal configurations of maxillary first premolars in 22 countries using two classification systems: a multinational cross-sectional study. Sci Rep. 2025;15(1):19290. 40456872 10.1038/s41598-025-02669-6 PMC12130479

[32] Ok E, Altunsoy M, Nur BG, Aglarci OS, Çolak M, Güngör E. A cone-beam computed tomography study of root canal morphology of maxillary and mandibular premolars in a Turkish population. Acta Odontol Scand. 2014;72(8):701–6. 24832561 10.3109/00016357.2014.898091

[33] Fernandes M, de Ataide I, Wagle R. C-shaped root canal configuration: A review of literature. J Conservative Dentistry. 2014;17(4):312–9. 10.4103/0972-0707.136437 PMC4127687 25125841

[34] Kato A, Ziegler A, Higuchi N, Nakata K, Nakamura H, Ohno N. Aetiology, incidence and morphology of the C-shaped root canal system and its impact on clinical endodontics. Int Endod J. 2014;47(11):1012–33. 24483229 10.1111/iej.12256 PMC4258081

[35] Martin G, Arce Brissón G, Chen B, Noemí de Caso C, Boetto AC, Jacobo MI, et al. Root dentine thickness in C-shaped lower second molars after instrumentation: A CBCT and micro‐CT study. Australian Endodontic J. 2021;47(2):122–9. 10.1111/aej.12509 33729636

[36] Yousefi F, Mohammadi Y, Shokri E. Prevalence of c-shaped canal morphology in premolar and molar teeth assessed by cone-beam computed tomography: systematic review and meta-analysis. BMC Oral Health. 2025;25(1):1657. 41126141 10.1186/s12903-025-06946-8 PMC12542057

[37] Fenelon T, Parashos P. Prevalence and morphology of C-shaped and non‐C‐shaped root canal systems in mandibular second molars. Aust Dent J. 2022;67(S1):S65–75. 35716050 10.1111/adj.12925

[38] von Zuben M, Martins JNR, Berti L, Cassim I, Flynn D, Gonzalez JA, et al. Worldwide Prevalence of Mandibular Second Molar C-Shaped Morphologies Evaluated by Cone-Beam Computed Tomography. J Endod. 2017;43(9):1442–7. 28734652 10.1016/j.joen.2017.04.016

[39] Guo Q, Wang Q, Yang Y, Guo D. Root and root canal morphology of mandibular second permanent molars in the Gansu province population: A CBCT study. Australian Endodontic J. 2022;49(S1):27–32. 10.1111/aej.12692 36125937

[40] Yang L, Han J, Wang Q, Wang Z, Yu X, Du Y. Variations of root and canal morphology of mandibular second molars in Chinese individuals: a cone-beam computed tomography study. BMC Oral Health. 2022;22(1):274. 35790917 10.1186/s12903-022-02299-8 PMC9258086

[41] Wang Y, Guo J, Yang H-B, Han X, Yu Y. Incidence of C-shaped root canal systems in mandibular second molars in the native Chinese population by analysis of clinical methods. Int J Oral Sci. 2012;4(3):161–5. 22836759 10.1038/ijos.2012.42 PMC3464989

[42] Pataer M, Abulizi A, Jumatai S, Zhang X, Zhang X, Zhao J. C-shaped canal configuration in mandibular second molars of a selected Uyghur adults in Xinjiang: prevalence, correlation, and differences of root canal configuration using cone-beam computed tomography. BMC Med Imaging. 2025;25(1):116. 40217193 10.1186/s12880-025-01640-1 PMC11987455

[43] Senan E, Alhadainy H, Madfa AA. Root and Canal Morphology of Mandibular Second Molars in a Yemeni Population: A Cone-beam Computed Tomography. Eur Endodontic J. 2021;6(1):72–81. 10.14744/eej.2020.94695 PMC8056815 33531451

[44] Lake S, Iwanaga J, Kikuta S, Oskouian RJ, Loukas M, Tubbs RS. The Incisive Canal: A Comprehensive Review. Cureus. 2018;10(7):e3069. 30280065 10.7759/cureus.3069 PMC6166911

[45] Panda M, Shankar T, Raut A, Dev S, Kar A, Hota S. Cone beam computerized tomography evaluation of incisive canal and anterior maxillary bone thickness for placement of immediate implants. J Indian Prosthodontic Soc. 2018;18(4):356-63. 10.4103/jips.jips_167_18 PMC6180735 30449964

[46] Gönül Y, Bucak A, Atalay Y, Beker-Acay M, Çalişkan A, Sakarya G, et al. MDCT evaluation of nasopalatine canal morphometry and variations: An analysis of 100 patients. Diagn Interv Imaging. 2016;97(11):1165–72. 26797526 10.1016/j.diii.2015.11.012

[47] Costa EDd, Nejaim Y, Martins LAC, Peyneau PD, Ambrosano GMB, Oliveira ML. Morphological Evaluation of the Nasopalatine Canal in Patients With Different Facial Profiles and Ages. J Oral Maxillofac Surg. 2019;77(4):721–9. 30597135 10.1016/j.joms.2018.11.025

[48] Daimaruya TTI, Nagasaka H, Umemori M, Sugawara J, Mitani H. Effects of Maxillary Molar Intrusion on the Nasal Floor and Tooth Root Using the Skeletal Anchorage System in Dogs. Angle Orthod. 2003;73(2):158–66. 12725372 10.1043/0003-3219(2003)73<158:EOMMIO>2.0.CO;2

[49] Rao JBTP, Pulla A, Kumar N, Patil SC, Tiwari I. Radiographic Assessment of Anatomy of Nasopalatine Canal for Dental Implant Placement: A Cone Beam Computed Tomographic Study. J Contemp Dent Pract. 2018;19(3):301–5. 29603703

[50] Connelly KJ, Larson EA, Marks DL, Klein RF. Neonatal Estrogen Exposure Results in Biphasic Age-Dependent Effects on the Skeletal Development of Male Mice. Endocrinology. 2015;156(1):193–202. 25330099 10.1210/en.2014-1324 PMC4272395

[51] Firincioglulari M, Orhan K. Morphological Variations of the Nasopalatine Canal in the North Cyprus Population: A Cone Beam Computed Tomography Study. Med Sci Monit. 2024;30:e944868. 38943242 10.12659/MSM.944868 PMC11305068

[52] Assoratgoon I, Wan B, Tenkumo T, Sato T, Kawata T, Hardani Putra R, et al. Three-dimensional in vivo and finite element analyses of peri-implant bone remodeling after superstructure placement. J Prosthet Dent. 2025;133(6):1535–44. 39909738 10.1016/j.prosdent.2025.01.015

[53] Botermans A, Lidén A, de Carvalho Machado V, Chrcanovic BR. Immediate Implant Placement in the Maxillary Aesthetic Zone: A Cone Beam Computed Tomography Study. J Clin Med. 2021;10(24):5853. 34945150 10.3390/jcm10245853 PMC8708737

[54] Rao P, jing J, Fan Y, Zhou C. Spatiotemporal cellular dynamics and molecular regulation of tooth root ontogeny. Int J Oral Sci. 2023;15(1):50. 38001110 10.1038/s41368-023-00258-9 PMC10673972

[55] Nagata M, Gadhvi GT, Komori T, Arai Y, Manabe H, Chu AKY, et al. Wnt-directed CXCL12-expressing apical papilla progenitor cells drive tooth root formation. Nat Commun. 2025;16(1):5510. 40595672 10.1038/s41467-025-61048-x PMC12216879

[56] Yang S, Choi H, Kim TH, Jeong JK, Liu Y, Harada H, Cho ES. Cell dynamics in Hertwig’s epithelial root sheath are regulated by β-catenin activity during tooth root development. J Cell Physiol. 2020;236(7):5387–98. 33377198 10.1002/jcp.30243 PMC8048837

[57] Burse A, Mahapatra J, Reche A, Awghad SS. Uncovering the Enigma of the C-shaped Root Canal Morphology. Cureus. 2024;16(6):e61883. 38975401 10.7759/cureus.61883 PMC11227744

[58] Seo H, Kim J, Hwang JJ, Jeong H-G, Han S-S, Park W, et al. Regulation of root patterns in mammalian teeth. Sci Rep. 2017;7(1):12714. 28983077 10.1038/s41598-017-12745-1 PMC5629201

[59] He M, Wang P, Li B, Wang Y, Wang X, Bai D, Guo Y. Rodent incisor and molar dental follicles show distinct characteristics in tooth eruption. Arch Oral Biol. 2021;126:105117. 33845260 10.1016/j.archoralbio.2021.105117

[60] Mu H, Liu X, Geng S, Su D, Chang H, Li L, et al. Epithelial Bone Morphogenic Protein 2 and 4 Are Indispensable for Tooth Development. Front Physiol. 2021;12:660644. 34483952 10.3389/fphys.2021.660644 PMC8415269

[61] Sadier A, Anthwal N, Krause AL, Dessalles R, Lake M, Bentolila LA, et al. Bat teeth illuminate the diversification of mammalian tooth classes. Nat Commun. 2023;14(1):4687. 37607943 10.1038/s41467-023-40158-4 PMC10444822

[62] Sagai T, Amano T, Maeno A, Kiyonari H, Seo H, Cho S-W, Shiroishi T. SHH signaling directed by two oral epithelium-specific enhancers controls tooth and oral development. Sci Rep. 2017;7(1):13004. 29021530 10.1038/s41598-017-12532-y PMC5636896

[63] Gomes BPFAAE, Kishen A. Irrigants and irrigation activation systems in Endodontics. Braz Dent J. 2023;34(4):1–33. 37909632 10.1590/0103-6440202305577 PMC10642269

[64] Dashti M, Ghadimi N, Yalcın TY, Zahmatkesh H, Orhan K, Rankouhi SI, et al. Three-Dimensional Filling Quality of Cold Lateral vs Warm Vertical Condensation: A Micro-CT and CBCT-Based Systematic Review and Meta-Analysis. Eur Endodontic J. 2025;10(6):479–87. 10.14744/eej.2025.22599 PMC12686864 41685848

[65] Candemil AP, Salmon B, Vasconcelos KF, Oenning AC, Jacobs R, Freitas DQ, et al. Cone beam CT optimisation for detection of vertical root fracture with metal in the field of view or the exomass. Sci Rep. 2021;11(1):19155. 34580339 10.1038/s41598-021-98345-6 PMC8476605

[66] Carney KA, Colloc TNE, Kilgariff JK. Management of rarely seen internal tunnelling root resorption associated with a maxillary permanent incisor. Br Dent J. 2024;236(12):955–61. 38942857 10.1038/s41415-024-7504-7 PMC11213701

[67] ElShebiny T, Abdelrauof D, Elattar M, Motro M, Retrouvey JM, El-Dawlatly M, et al. A deep learning model for multiclass tooth segmentation on cone-beam computed tomography scans. Am J Orthod Dentofac Orthop. 2025;168(2):191–8. 10.1016/j.ajodo.2025.02.014 40186597

[68] Tarakçı ÖD, Kış HC, Amasya H, Öztürk İ, Karahan E, Orhan K. Radiomics-Based Diagnosis in Dentomaxillofacial Radiology: A Systematic Review. J Imaging Inf Med. 2024;38(4):2428–61. 10.1007/s10278-024-01307-3 PMC12344034 39528882

[69] Kamburoğlu K. Trends in dentomaxillofacial radiology. World J Radiol. 2025;17(1):97255. 39876885 10.4329/wjr.v17.i1.97255 PMC11755909

